# Retraction: Resveratrol protects against apoptosis induced by interleukin-1β in nucleus pulposus cells via activating mTOR/caspase-3 and GSK-3β/caspase-3 pathway

**DOI:** 10.1042/BSR-20202019_RET

**Published:** 2020-08-18

**Keywords:** Apoptosis, Interleukin-1, Intervertebral disc, Nucleus pulposus cells, Resveratrol

This article is being retracted from Bioscience Reports at the request of the authors following receipt of a notification from a reader, alerting the authors to common features in the FACS plots of Figure 1.

The authors have been unable to replicate the results, hence felt it was necessary to retract the paper. The Editorial Board agrees to the retraction.

